# Pattern and Risk Factors of Central Compartment Lymph Node Metastasis in Papillary Thyroid Cancer: A Prospective Study from an Endocrine Surgery Centre

**DOI:** 10.1155/2012/436243

**Published:** 2011-09-29

**Authors:** Sudhi Agarwal, Gyan Chand, Sushila Jaiswal, Anjali Mishra, Gaurav Agarwal, Amit Agarwal, A. K. Verma, S. K. Mishra

**Affiliations:** ^1^Department of Endocrine Surgery, Sanjay Gandhi Postgraduate Institute of Medical Sciences, Lucknow 226014, India; ^2^Department of Pathology, Sanjay Gandhi Postgraduate Institute of Medical Sciences, Lucknow 226014, India

## Abstract

Lymphatic metastasis in papillary thyroid cancer (PTC) is eminent; however, the extent of central compartment lymph nodes dissection (CCD) is controversial and requires the knowledge of pattern and risk factors for central compartment lymph nodes metastasis (CCM). We did a prospective study of 47 cases with PTC who underwent total thyroidectomy (TT) with CCD with/without lateral lymph nodes dissection (LND). Clinicopathological profile including CCM as ipsilateral and contralateral was documented. On histopathology, the mean tumour size was 3.57 ± 2.42 cm 59.6% had CCM, which was bilateral in the majority (60.72%). The tumour-size was the most important predictor for lymph nodes metastasis-(*P=0.018*) whereas multicentricity-(*P=0.002*) and ipsilateral CCM-(*P=0.001*) were the predictors for contralateral CCM. The long-term morbidity of CCD done in primary setting is comparable with TT-alone. Bilateral CCD should be done with thyroidectomy in PTC, otherwise the risk of residual diseases and subsequent recurrence is high. The long-term morbidity is comparable in experienced hands.

## 1. Introduction

Papillary thyroid cancer (PTC) is the most common thyroid malignancy with the predilection for lymphatic spread [1]. Like any other head and neck malignancy, the lymphatic spread of PTC is supposed to follow a sequential pattern with the central compartment or level VI lymph nodes being first to involve [2–4], which besides containing the pre- and paratracheal lymph nodes, also contains the parathyroid glands and recurrent laryngeal nerves on either side, which are prone to injury while dissecting the central compartment lymph nodes [4]. The central compartment lymph nodes dissection (CCD) in PTC has the advantages of complete clearance of the disease, thereby reducing the chances of recurrence and the subsequent morbidity of reoperation, also it provides the nodes for exact nodal staging to plan further adjuvant therapy and prognosticate the patient [5, 6]. However, it is associated with increased risk of hypoparathyroidism and recurrent laryngeal nerve palsy [7]. Therefore for high-risk with clinically involved nodes the routine CCD is acceptable; however, it is controversial for low-risk, clinically uninvolved nodes [8], with some advocating for [9, 10] and some against [11] routine bilateral clearance while a third group of surgeons adopted a midway, by dissecting the ipsilateral side only, thus sparing the contralateral parathyroid glands and recurrent laryngeal nerve [12–14]. The extent of lymphadenectomy in PTC is still controversial and requires the knowledge of pattern of central compartment lymph nodes metastasis. Therefore, we aim our study to find out the pattern and risk factors of central compartment lymph node metastasis (CCM), and the morbidity of CCD.

## 2. Material and Method

We did a prospective study of all patients with papillary thyroid cancer, who underwent total thyroidectomy (TT) with CCD as a primary surgery from September 2008 till November 2010 at Department of Endocrine Surgery, Sanjay Gandhi Postgraduate Institute of Medical Sciences, Lucknow, India. The study was approved by department review board, written and informed consent was taken from the patients, as per the department protocol. Preoperatively, besides a detailed history and examination, we did the ultrasound neck and fine-needle aspiration cytology of the thyroid nodules and any suspicious cervical lymph nodes, and a preoperative clinical staging based on 6th AJCC [15] was done. Final staging was done after the histopathology report (p-TNM). We excluded patients with missed initial diagnosis of PTC who did not undergo CCD, reoperative surgeries (like completion thyroidectomy and locoregional recurrence), and concomitant hyperparathyroidism. “Central compartment” is defined as an area that is bounded superiorly by the hypoid bone, inferiorly by the innominate vein, and on either side by the carotid sheaths [16]. We did the thyroidectomy with standard technique of capsular dissection. The thyroid along with the central compartment lymph nodes was removed en bloc in all the cases and the fibrofatty tissue was divided in midline and sampled as right and left paratracheal lymph nodes [9]. Formal lateral lymph nodes dissection involving the level II, III, and IV were done in patients with proven lateral lymph nodes metastasis. We routinely identified all the parathyroid glands, external branch of superior laryngeal nerves and recurrent laryngeal nerves and tried to save them in situ. Any at-risk gland was autotransplanted in sternocleidomastoid muscle. We grossed the specimen in operation theatre and in pathology department under supervision of a single pathologist (SJ) involved in the study. Along with detailed grossing of thyroid, the individual lymph nodes from lymph nodes basins were also separated, counted, and embedded. Following processing 3 *μ*m thick sections were examined under microscope. For the purpose of description, the term “ipsilateral” was designated to the side with largest tumor size [9]. The complications were assessed in immediate postoperative period and 6 months following surgery and compared with a group of benign thyroid disorders, who underwent total thyroidectomy alone in primary setting during the same period. “Hypocalcemia” was defined as corrected serum calcium ≤8 mg/dL with or without clinical features of hypocalcemia. Any patient with significant postoperative hoarseness underwent vocal cord examination by indirect laryngoscopy to document recurrent laryngeal nerve (RLN) palsy. Hypocalcemia and RLN palsy are termed as temporary when it is resolved within 6 months of surgery. Patients with hypocalcemia were managed with oral/parenteral calcium and vitamin D, as per department protocol [17–19]. Following 6 months after surgery we did the serum parathyroid hormone analysis in any patient with persistently low serum calcium and requiring calcium and vitamin D support, thereby documenting permanent hypoparathyroidism. Similarly in all the patients with RLN palsy, vocal cord assessment was done at 6 months following surgery to document permanent RLN paralysis. 

### 2.1. Statistical Analysis

Analysis was done by the SPSS 17. We used frequency, percentages, mean ± standard deviation, range, Student's *t*-test, chi-square test, and Fischer exact test, wherever applicable. Univariate and multivariate analysis is done using logistic regression.

## 3. Results

As shown in Table 1 at the end of study 47 cases were included. The mean age was 35.79 ± 12.65 years with female preponderance (31 : 16). Majority were presented with solitary thyroid nodule (42/47). 4/47 cases presented with lateral lymph node metastasis with occult PTC. Majority were node negative (29/47) on clinical examination and imaging. CCD was done in all cases. 19/47 patients underwent lateral lymph nodes dissection also Table 2. Histopathology of thyroid showed, mean tumor size 3.57 ± 2.42. Classical variant was present in 35/47, other variants being follicular, oncocytic variant [20], and tall cell. Tumor was bilateral in 9/47 cases, multicentric in 18/47, and with extra thyroidal extension in 13/47 cases. Overall lymph node metastasis was present in 30/47. Out of which 28/47 cases had CCM. Majority had bilateral central compartment lymph nodes metastasis (17/47), with isolated ipsilateral CCM in 9/47 and isolated contralateral CCM in 2/47. All patients with lateral lymph nodes dissection (19/47) had metastatic lateral lymph nodes on histopathology. Isolated ipsilateral LLNM was present in 15/19, bilateral LLNM in 3/19, and isolated contralateral LLNM in 1/19 cases. Skip lateral metastasis, skipping the central compartment was present in 2/19 and skip contralateral CCM, skipping the ipsilateral CCLN was present in 2/45 cases. At the end of study, 3 cases were upstaged from clinically node negative to histopathologically proven node positive (2 cases with CCM and one patient with CCM and lateral LNM). The univariate analysis was done to find out the association of various known risk factors like age, age group (≤45 years), sex, tumor size, pathological tumor staging, tumor subtypes, multicentricity, bilaterality, and extrathyroidal extension (Table 3). We did find a significant association of tumor size with the CCM (4.2 ± 2.45 versus 2.58 ± 2.02; *P* = 0.018). Similarly, ipsilateral CCM was significantly associated with primary tumor size (4.4 ± 2.4 versus 2.5 ± 2.0; *P* = 0.013), and contralateral CCM was significantly associated with multicentricity (36.8% versus 18.5%; *P* = 0.003) and ipsilateral CCM (89.5% versus 33.3%; *P* = 0.000). Multivariate analysis showed ipsilateral CCM metastasis as a significant risk factor for contralateral CCM (*P* = 0.02). In our series, 42 patients initially presented with solitary thyroid nodule confined to one lobe only, but on final histopathology, 8/42 were found to have tumor foci in both the lobes (bilateral tumors) and 16/42 was multicentric. Two patients in our series had skip metastasis to contralateral CCM, skipping the ipsilateral CCM, one of them was a 40-year-old male, who presented with right lateral lymph nodes metastasis with occult PTC, underwent TT with CCD with right MRND, and on final histopathology was detected to have 0.5 cm tumor in right lobe thyroid with multicentricity and bilaterality; second patient, was a 44-year-old male with clinically evident solitary thyroid nodule with absent nodes, underwent TT with CCD, and on histopathology was found to have 4 cm tumor with no evidence of multicentricity and normal opposite lobe. Similarly, for skip lateral metastasis that is skipping the CCM, out of two patients, one was a 33-year-old male with lateral lymph nodes metastasis with occult PTC, histopathology showed a 0.4 cm tumor, with multicentricity with normal opposite lobe; the second patient was a 62-year-old lady, who presented with solitary thyroid nodule with lateral lymph nodes metastasis, the histopathology again revealed a 0.3 cm tumor with no multicentricity and normal opposite lobe; however, the extrathyroidal extension was present. All the above-mentioned cases had classical variant of PTC. All 47 cases underwent whole-body radioactive iodine scan 4 to 6 weeks following thyroxin withdrawal, and radioactive remnant ablation was done in 41/47 patients. All patients received suppressive L-thyroxin therapy. The morbidity was compared with age- and sex-matched group of benign thyroid disorders operated during the same period that underwent total thyroidectomy alone (Table 4). In our series, though the rate of inadvertent parathyroidectomy, temporary hypocalcaemia, and temporary RLN palsy was significantly high in group with CCD (*P* < 0.05), the long-term morbidity, that is, rate of permanent RLN palsy and hypoparathyroidism was comparable to those with total thyroidectomy alone.

## 4. Discussion

PTC has propensity for lymphatic spread [1]. The metastasis is found in 20–50% of lymph nodes examined by conventional pathologic examination whereas the rate of micrometastasis has been found to be much higher in clinically node negative-cases [21]. The lymph node metastasis in papillary thyroid cancer has been found to have significant impact on disease-free and overall survival of patients [22, 23]. Giles et al. [24] in their retrospective series of 343 patients with thyroidectomy but without CCD found a locoregional recurrence rate of 6% with a median followup of 9 ± 4 years, where central compartment lymph nodes were involved in 6 recurrent cases. Similarly, Moo et al. [5], in their series, during a mean followup of 3.1 years, reported increased locoregional recurrences in the group undergoing total thyroidectomy alone (16.7%) compared to those with prophylactic central lymph nodes dissection (4.4%). Similarly, Ito et al. [25] reported 10-year disease-free survival (DFS) of 97% with elective lymph nodes dissection in low- risk PTC even without radio-iodine treatment. On the other hand Rosenbaum and McHenry [7], reported, no significantly reduced recurrence rate with central neck dissection, but an increased risk of temporary hypocalcemia, compared with no central cervical lymph nodes dissection group. The rationale behind the routine dissection of central compartment lymph nodes in preoperatively diagnosed cases of PTC with clinically uninvolved lymph nodes is manifold; first, the PTC has the tendency for lymphatic spread, which tends to follow a sequential pattern with ipsilateral paratracheal followed by contralateral paratracheal and ipsilateral lateral cervical lymph nodes [3, 4]; secondly, it provides accurate tumor staging to facilitate accurate prognosis and adjuvant therapy [6]; lastly, the long-term morbidity of CCD in primary setting in experienced hands is comparable with total thyroidectomy alone [5, 9] and even though if it is higher [10, 26], it is still lower than the morbidity of central compartment lymph nodes dissection (CCD) done in reoperative setting [27]. Roh et al. [28], in their prospective series of 45 patients with recurrent PTC detected 86.7% recurrences in central compartment, with high rates of temporary and permanent complications with reoperation. There are few reports documenting no increased risk of complications following reoperative surgery compared to CCD done in primary setting [29–31]; however, there was also no benefit of reoperative surgery as well. The concern, whether we should remove the ipsilateral central compartment lymph nodes based on obvious tumor location is also being studied by many investigators, [9, 12–14] and is also controversial. Sadowski et al. [9] in their study reported a mean tumor size of 1.26 cm, 25.5% rate of contralateral CCM. Similarly Roh et al. [32], in their prospective series of 52 cases with lateral LNM, had 8.9% of contralateral CCM. Likewise, Ito et al. [33], reported 27.3% of contralateral paratracheal lymph node metastasis, the frequency of which was low for tumors ≤1 cm (9.8%), but increased with tumor size. They also reported significantly decreased DFS due to contralateral paratracheal LNM on univariate analysis. Koo et al. [34] reported 6.7% contralateral CCM with unilateral PTC, the rate of which was significantly increased with maximum tumor diameter >1 cm, lymphovascular invasion and ipsilateral lymph nodes metastasis. In their other study [35], they found 34.3% of occult contralateral lymph nodes involvement with unilateral lymph nodes metastasis in lateral neck, which on multivariate analysis was found to be significantly associated with multifocality, ipsilateral CCM, and lateral LNM. The rate of micrometastasis to ipsilateral lateral lymph nodes also increases with tumor size, and its prophylactic dissection has been recommended by some along with ipsilateral CCD [16]; however, it is still not recommended [8] because those compartments are not violated during thyroidectomy, and, therefore, their surgery at a later date will not pose for increased risk of complications. 

The sensitivity of preoperative ultrasound (US) in detecting pathological cervical lymph nodes is 62% with the sensitivity lower for cervical lymph nodes than for lateral lymph nodes (55% versus 65%) [36], and is higher for central compartment lymph nodes following thyroidectomy than with thyroid (90.4% versus 83.5%) [37]. Therefore, it is not a reliable indicator of node negativity and treatment decision cannot be based solely on it.

In our study, for a mean primary tumor size of 3.57 ± 2.42 cm, 59.6% of the patients had central cervical lymph nodes metastasis, which agrees with other studies. Unlike Gimm et al. [3], in our series, the majority of the metastasis (60.72%) involved bilateral central cervical lymphnodes followed by isolated ipsilateral central cervical lymph nodes (32.14%). 3/29 (10.34%) cases with apparently node negative disease were found to have central compartment lymph nodes metastasis was present on final histopathology. Similarly for the thyroid, out of 42 cases with apparently solitary thyroid nodule confined to one lobe, 8 (19.5%) were found to be bilateral and 16 (38.1%) were multicentric. Koo et al. [38], found 16.7% cases with occult contralateral PTC with apparently unilateral papillary microcarcinoma; those cases were missed on preoperative imaging due to their small size. Various risk factors for central compartment lymph node metastasis were analyzed by investigators with variable results [39–41]. Majority found a significant association of lymph nodes metastasis with tumour size and multicentricity [33–35], other factors being age, gender, extracapsular invasion vascular invasion, and lymphovascular invasion. We did find the tumour size a significant predictor of central cervical lymph nodes metastasis, and ipsilateral central cervical lymph nodes metastasis, whereas multicentricity and ipsilateral central lymph nodes metastasis were significant predictors of contralateral central cervical lymph nodes metastasis. Koo et al. [34, 35] also had similar observations, which imply that, for a primary tumor, apparently confined to one lobe, but with ipsilateral central or lateral metastatic lymph nodes, the risk of contralateral central lymph nodes metastasis is proportionately high and their removal is required for complete disease clearance. Regarding size of the tumour, though the risk of lymph node metastasis increases with tumour size, the papillary microcarcinoma (PMC) was not exempt of lymph nodal metastasis in our series. We had 5 out of 8 (62.5%) cases with PMC, presented with lateral LNM, in which 3 had associated CCM and 2 cases had skip metastasis, which is contrary to the studies which consider PMC as an indolent disease with low risk for nodal metastasis [42]. It may probably be answered by molecular mutations analysis [43].

Many investigators advocate the use of adjuvant radioactive iodine ablation as an option to take care of residual microscopic disease; however, it has drawbacks of complications and patient's discomfort, and also it may not be readily available at many centres like in India, or may be contraindicated, like in children [44], or may be legally restricted like in Japan. Also the literature does not support its role to decrease the locoregional recurrence, compared to surgery [44, 45]. Excellent prognosis has been reported by Ito et al. [25], in low-risk PTC who underwent thyroidectomy with elective bilateral clearance of central lymph nodes without radioactive iodine therapy. 

Like other studies [9, 21], though we had higher rate of inadvertent parathyroidectomy, temporary hypocalcemia, and RLN palsy (*P* = 0.000) in patients who underwent central lymph nodes dissection compared to total thyroidectomy alone, the long-term complications were comparable in both the groups. The risk of reoperative surgery is technically always challenging and associated with higher morbidity [29–31], with no benefit over surgery done in primary setting. In countries like India, where the resources are scarce, patients are not medically insured, radioactive iodine is not readily available, and the regular followups are difficult, it is hard to motivate the patients for redo surgeries, rather patients are more inclined for complete disease removal in primary setting itself. 

The strong factors with our study is its prospective well-controlled design, which eliminates as much biases as possible; however, like any studies, it has limitations due to its small sample size, shorter followups and the nonrandomized nature for comparing the morbidity. But on comparing with other prospective studies, we have included considerably more number of patients per year, and we hope to bring more robust data in coming years.

## 5. Conclusion

The rate of central compartment lymph node metastasis is high in PTC. Tumour size is an important risk factor for CCM. Multicentricity and ipsilateral lymph node metastasis are risk factors for contralateral CCM. Bilateral CCM is most frequent rather than ipsilateral CCM. The long-term morbidity of bilateral CCD done in primary setting is comparable with total thyroidectomy alone, and is safe in the hands of experienced endocrine surgeons.

## Figures and Tables

**Table 1:** Demographic profile of the patients; *n* = 47 (% age).

Mean age ± SD (range) years	35.79 ± 12.65 (9–69)
Age group	
(i) ≤45 years	40 (85.1)
(ii) >45 years	7 (14.9)
Sex (male : female)	31 : 16
Clinical presentation	
(i) Solitary thyroid nodule	42 (89.4)
(ii) Multinodular goiter (MNG)	2 (4.3)
(iii) MNG with dominant nodule	3 (6.4)
Occult papillary thyroid cancer	4 (8.5
Functional status	
(i) Euthyroid	41 (87.2)
(ii) Hypothyroid	6 (12.8)
Clinical nodal staging (TNM-classification)	
(i) cN0	29 (61.7)
(ii) cN1b	18 (38.3)
Surgical procedure	
(i) TT + B/L CCD	28 (59.6)
(ii) TT + B/L CCD + U/L MRND	13 (27.7)
(iii) TT + B/L CCD + B/L MRND	4 (8.5)
(iv) TT + U/L CCD + U/L MRND	2 (4.3)

**Table 2:** Histopathological features; *n* = 47 (% age).

Thyroid	
Mean tumor size (cm) ± SD (range)	3.57 ± 2.42 (0.3–12)
Tumor staging	
(i) T1	4 (8.5)
(ii) T2	19 (40.4)
(iii) T3	11 (23.4)
(iv) T4	13 (27.7)
Papillary microcarcinoma	8 (17)
PTC subtypes	
(i) Classical variant (CV)	35 (74.5)
(ii) Follicular variant (FV)	8 (17)
(iii) Oncocytic variant (OV) [20]	1 (2.1)
(iv) Tall cell variant (TCV)	3 (6.4)
Laterality	
(i) Right side	29 (61.7)
(ii) Left side	18 (38.3)
Multicentricity	18 (38.3)
Bilaterality	9 (19.1)
Extrathyroidal extension	13 (27.7)

Lymph nodes	

Overall lymph nodes metastasis	30 (63.8)
Overall central compartment lymph nodes metastasis (CCM)	28 (59.6)
(i) Bilateral CCM	17 (60.72)
(ii) Isolated ipsilateral CCM	9 (32.14)
(iii) Isolated contralateral CCM	2 (7.14)
Overall lateral compartment lymph nodes metastasis (LLNM; *n* = 19)	19 (100)
(i) Ipsilateral LLNM	15 (78.95)
(ii) Bilateral LLNM	3 (15.8)
(iii) Contralateral LLNM	1 (5.3)
Final pathological nodal staging (TNM-classification)	
(i) pN0	25 (53.2)
(ii) pN1a	3 (6.4)
(iii) pN1b	19 (40.4)
Skip lateral metastasis (*n* = 19)	2 (10.5)
Skip contralateral central lymph node metastasis (*n* = 47)	2 (4.3)

**Table 3:** Univariate analysis of central compartment lymph nodes metastasis (CCM), ipsilateral CCM and contralateral CCM with known risk factors.

	CCM			Ipsilateral CCM			Contralateral CCM		
	Present *n* = 28	Absent *n* = 19	*P* value	Present *n* = 26	Absent *n* = 21	*P* value	Present *n* = 19	Absent *n* = 28	*P* value
Age (years)	34.96 ± 14.41	37 ± 9.74	0.594	34.42 ± 14.83	37.48 ± 9.38	0.410	34.68 ± 15.77	36.67 ± 11.03	0.602
Age group									
≤45 years	24	16	1.000	22	18	1.000	16	23	0.928
>45 years	4	3		4	3		3	4	
Sex									
Male	9	7	0.763	76	9	0.355	7	8	0.608
Female	19	12		19	12		12	19	
Tumor size (cm)	4.2 ± 2.45	2.58 ± 2.02	0.018*	4.4 ± 2.4	2.5 ± 2.0	0.013*	4.02 ± 2.23	3.38 ± 2.5	0.378
Tumor staging									
T1	2	2		1	3		2	1	
T2	8	11	0.326	8	11	0.760	5	14	0.324
T3	8	3		8	3		5	6	
T4	10	3		9	4		7	6	
Tumor subtype									
Classical variant	22	13		20	15		14	20	
Follicular variant	3	5	0.580	3	5	0.619	2	6	0.434
Oncocytic variant	1	0		1	0		1	0	
Tall cell variant	2	1		2	1		2	1	
Multicentricity	13	5	0.169	12	6	0.245	12	5	0.002*
Bilaterality	6	3	0.631	5	4	1.000	6	3	0.085
Extrathyroidal extension	10	3	0.144	10	3	0.102	7	5	0.170
Ipsilateral CCM	—	—	—	—	—	—	17	9	0.001*

**Table 4:** Comparison of morbidity following total thyroidectomy with CCD (*n* = 47); with total thyroidectomy alone (benign thyroid disorders; *n* = 130).

	Total thyroidectomy with CCD; *n* = 47 (% age)	Total thyroidectomy alone; *n* = 130 (% age)	*P* value
Inadvertent parathyroidectomy	13 (27.7)	0	
Temporary hypocalcemia	41 (87.2)	67 (51.5)	0.000*
Temporary RLN palsy	8 (17)	9 (6.9)	0.044*
Permanent hypoparathyroidism	1 (2.1)	3 (2.3)	0.458
Permanent RLN palsy	1 (2.1)	1 (0.8)	0.419
